# Design of Zeolitic Imidazolate Framework-8-Functionalized Capacitive Micromachined Ultrasound Transducer Gravimetric Sensors for Gas and Hydrocarbon Vapor Detection

**DOI:** 10.3390/s23218827

**Published:** 2023-10-30

**Authors:** Mindaugas Dzikaras, Dovydas Barauskas, Donatas Pelenis, Gailius Vanagas, Marius Mikolajūnas, Jingming Shi, Jonas Baltrusaitis, Darius Viržonis

**Affiliations:** 1 Panevėžys Faculty of Technology and Business, Kaunas University of Technology, 37164 Panevėžys, Lithuania; dovydas.barauskas@ktu.lt (D.B.); donatas.pelenis@ktu.lt (D.P.); gailius.vanagas@ktu.lt (G.V.); marius.mikolajunas@ktu.lt (M.M.); darius.virzonis@ktu.lt (D.V.); 2 Department of Chemical and Biomolecular Engineering, Lehigh University, B336 Iacocca Hall, 111 Research Drive, Bethlehem, PA 18015, USA; jis223@lehigh.edu (J.S.); job314@lehigh.edu (J.B.)

**Keywords:** gravimetric, gas sensor, ZIF-8, CMUT, hydrocarbons

## Abstract

A capacitive micromachined ultrasound transducer (CMUT) was engineered and functionalized with zeolitic imidazolate framework-8 (ZIF-8) dispersed in a photoresist AZ1512HS (AZ) matrix to function as a gravimetric gas sensor. The sensor response was recorded in the presence of nitrogen, argon, carbon dioxide, and methane gases as well as water, acetylene, a propane/butane mixture, n-hexane, gasoline, and diesel vapors. The photoresist matrix alone was found to have a negligible response to all the gases and vapors, except for water vapor. No visible difference in sensor response was detected when switching from nitrogen to methane gas. However, a strong shift in the sensor resonance frequency was observed when exposed to higher hydrocarbons, ranging from 1 kHz for acetylene to 7.5 kHz for gasoline. Even longer-chain hydrocarbons, specifically kerosene and more so diesel, had a significantly reduced sensor frequency shift compared with gasoline. Sensors functionalized with a thin film of AZ+ZIF-8 demonstrated higher sensitivity in their response to a hydrocarbon molecular mass than without functionalization.

## 1. Introduction

Methane (CH_4_) and higher hydrocarbons are the main sources of heat, propulsion, and industrial energy. Naturally extracted hydrocarbons can have various impurities, such as carbon dioxide, and various reduced-sulfur compounds, some of which can be carcinogenic and toxic, while others can lower the energy density of the fuel or impede the transport logistics [1,2,3,4,5]. Furthermore, hydrocarbon fractionation is required for specific applications, such as in jet fuel, for optimal burning conditions and due to stringent environmental requirements [6]. Additionally, methane and higher hydrocarbons pose explosion and flammability hazards in natural gas or petroleum extraction and refinement as well as mining industries. Therefore, suitable testing and monitoring platforms for fuel composition and cleanliness are necessary.

Typically, gas sensors have a few types of operation. Popular non-dispersive infrared (NDIR) sensors have poor cross-selectivity due to their closely spaced mid-IR absorption footprints between 5 nm and 8 nm [7]. NDIRs can be improved by incorporating microelectromechanical systems (MEMS) or micro-optical systems that lead to better selectivity and the ability to detect multiple gases, but they are a more complex and expensive option. Examples of such systems are cavity ringdown spectroscopy [8], tunable diode laser absorption spectroscopy [9], and NDIRs with tunable band-pass filters [10,11]. A strategy of integrating several NDIRs with fixed optical filters for selective multiple gas detection is impractical due to the requirement of an individual optical element for each measurement channel. Fourier transform infrared spectroscopy (FTIR) and other commonplace laboratory-based techniques are too bulky and too expensive to be portable, mass-produced, and ubiquitous solutions. A polymer-coated Bragg grating sensor was recently demonstrated for simultaneous CO_2_ and water vapor detection; however, the CO_2_ time resolution was lacking [12].

Some metal–organic frameworks (MOFs) are excellent microporous adsorbents due to their ultrahigh specific surface and selective pore size. This allows their use for the adsorption of specific gases for storage purposes or filtration through MOF–compound membranes [13,14]. Zeolitic imidazolate framework-8 (ZIF-8), a zeolitic MOF, has been widely researched as the basis of such gas adsorbing and separation platforms [15,16]. It has been demonstrated to be a suitable material for nitrogen (N_2_)/CH_4_, propylene/propane, ethene/ethane, and other gas mixture separations [17,18,19,20,21]. With specific ZIF-8 modifications, its suitability for selective gas adsorption and permeability can be tailored [17,22].

ZIF-8 has already proven itself to be a flexible material for the differentiation, adsorption, and filtration of various gases. It has also shown potential as a material for the construction of sensing devices. Some gas sensors based on ZIF-8, such as the CH_4_ optical fiber-based sensor, have already been demonstrated [23], including as a chemo-resistive sensor [24]. This paper aimed to demonstrate the feasibility, characteristics, and versatility of a gravimetric MEMS capacitive micromachined ultrasound transducer (CMUT)-based ZIF-8-modified gas sensor. Successful gravimetric CMUT devices have been demonstrated before [25,26,27,28]. CMUTs are popular as a mass-sensing platform due to their sensitivity, ease of manufacture, scaling, and electronic response. In this paper, the authors micromachined and tested CMUT-based ZIF-8-functionalized sensor sensitivity to various gases with emphasis on functionality when switching between gases.

CMUT technology offers several advantages over other gravimetric transducers, such as fabrication flexibility, integration potential, higher mass sensitivity, and a higher signal-to-noise ratio [29,30]. The microscale fabrication technique allows for an extremely small footprint, unlocking its use in size-constrained applications. Excellent mass sensitivity and resolution can be achieved because CMUT structures have small membranes and can be designed for highly resonant frequencies and quality factors. Furthermore, CMUT arrays can be densely packed with individual elements, leading to reduced cross-talk between adjacent transducer elements. Integrating the elements into large arrays leads to a better SNR. The fabrication versatility of CMUTs can be included as another advantage as semiconductor fabrication techniques can be used, which offer precise control over the element size and geometry. This allows for customized transducer designs and integration with electronics on the same chip. Despite these advantages, CMUTs also have some limitations, including their complexity in fabrication and the potential for higher costs compared with traditional transducers. However, ongoing research and development efforts aim to address these challenges and further enhance the performance and affordability of CMUT technology.

## 2. Materials and Methods

### 2.1. CMUT-Based ZIF-8-Functionalized Sensor Manufacturing

**Figure 1:**
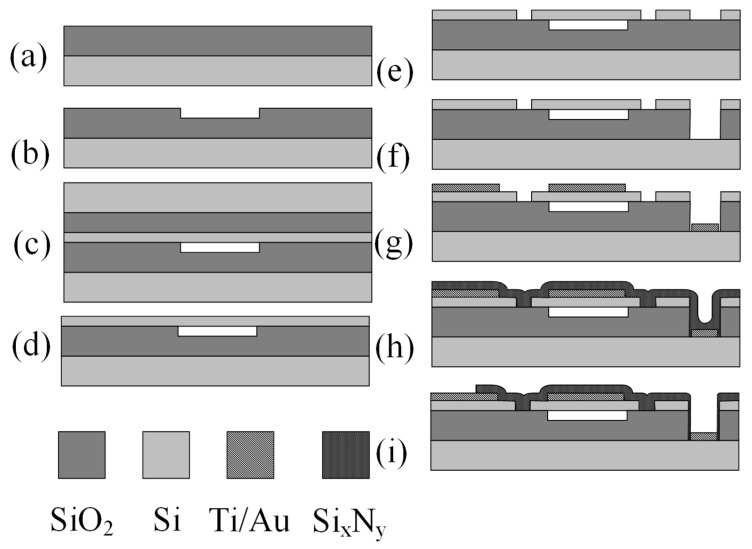
CMUT fabrication process using wafer-bonding technique: (**a**) oxidation; (**b**) cavity formation; (**c**) wafer bonding; (**d**) handle-wafer removal; (**e**) deep reactive ion etching; (**f**) ground pad opening formation; (**g**) top electrode formation and metallization; (**h**) passivation layer formation; (**i**) contact pad opening.

The CMUT devices were designed to have a nominal 14 MHz resonance frequency. The devices were manufactured using standard UV photolithography processes, which are shown in Figure 1 and described in previous works [26,31]. Briefly, using the first lithography step, the oxidized wafers (Figure 1a) were etched with a buffered oxide etchant to form cavities (Figure 1b). Then, a silicon-on-insulator (SOI) wafer with a device layer thickness of 2 µm was bonded (Figure 1c) on top. The handle wafer was removed by a chemical–mechanical polishing process and wet etching (Figure 1d). Another UV photolithography step was applied to the devices and membrane trenches were separated using a deep reactive ion etching process using the Oxford PlasmaPro system, which repeatedly combines isotropic silicon etching and passivation steps using a high-density plasma source and fast gas-switching capability (Figure 1e) [32]. Another UV lithography step created openings for the ground electrode (Figure 1f) with a reactive ion etching process. Then, the top and bottom electrodes were formed using a thin metal layer deposition CVD process (Figure 1g). Finally, a protective silicon nitride layer was formed on top using plasma-enhanced chemical vapor deposition (Figure 1h) and the last lithography step was performed to etch contact pad openings with reactive ion etching (RIE) (Figure 1i). In total, 5 photomasks were necessary to fully manufacture the CMUT devices. Lastly, the finished wafers were diced and the individual CMUT chips were separated and wire bonded to custom-printed circuit boards for ease of connection to the electronic and testing equipment.

### 2.2. ZIF-8 Synthesis

ZIF-8 was synthesized using a method described by Cao et al. [23]. First, 0.74 g of zinc nitrate hexahydrate (Merck) was dissolved in 10 mL of methanol (Merck) by shaking. Then, 0.41 g of 2-methylimidazole (Merck) was added, shaken, and the solution was left at room temperature for 24 h. Afterward, the solution was centrifuged at 978× *g* for 10 min, the supernatant was discarded, 10 mL of fresh methanol was added, and the precipitant was resuspended by ultrasonication for 10 min. This washing process was performed two more times, with the final resuspension performed in 2 mL of methanol.

### 2.3. Sensor Functionalization

Multiple CMUT devices were functionalized with different materials that included AZ1512HS (herein referred to as AZ) photoresist (MicroChemicals), ZIF-8 mixed with the AZ1512HS photoresist (herein referred to as AZ+ZIF-8), and graphene oxide (GOx, Merck). AZ was found to suspend ZIF-8 well and both were dissolvable in methanol. AZ and all of the prepared suspensions were kept at 4 °C in darkness.

**Figure 2:**
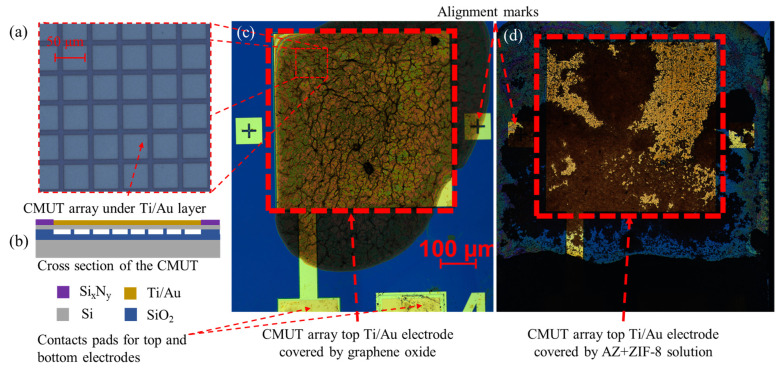
Micrographs of CMUT devices and their components. (**a**) Individual CMUT cells in an array visible on half-assembled CMUT devices (just before the bonding step). (**b**) A cross-section schematic and material indications of the individual CMUT cells. The schematic is not to scale with the rest of the images. (**c**) Micrographs of CMUT devices modified by drop-coating with 2 µL graphene oxide and (**d**) with 2 µL AZ+ZIF-8 solution.

During AZ functionalization, AZ was diluted 50-fold using methanol (Merck) and drop-coated on the CMUT active area. For AZ+ZIF-8 functionalization, the diluted AZ was mixed at a 1:1 ratio with the ZIF-8 solution (Figure 2d). For the additional methanol-diluted experiments, the prepared AZ+ZIF-8 solution was further diluted with methanol in 1:2, 1:4, 1:8, and 1:16 ratios (AZ+ZIF-8:methanol) to reduce the functional material mass. For the graphene oxide (GOx) modification, 2 mg/mL of the GOx solution (Merck) was diluted to 0.2 mg/mL and used. In all device functionalization cases, 2 µL of the solution was used to drop-coat the devices (Figure 2c). Functionalized devices were kept at a temperature of 21 °C, 40–50% humidity, and in complete darkness when not used for measurements.

### 2.4. Electronics Setup

The electronics used during the experiments were designed and assembled for 4 different channel resonance frequency measurements that could gather data for each modified CMUT device every couple of seconds. It was used together with a universal serial bus (USB) interface and custom MATLAB version R2022a update 2 (9.12.0.1956245) software for ease of data acquisition and analysis. In addition to the 4 channels, the electronics setup also had an integrated humidity and temperature sensor for reference measurements. A STM32F042 microcontroller was used to read the signal coming from all four channels and these channels were multiplexed to a Colpitts-type oscillator, while the frequency readings were obtained using the microcontroller’s high-speed counter input.

The analog signal coming from the oscillator circuitry was normalized for the microcontroller by an op-amp. The microcontroller then detected the rising edges of the input signal and for each detected rising edge, the controller increased an internal counter by one. Simultaneously, a fixed duration timer was set to trigger every 100 ms that stopped the counting process. The resonator’s frequency was calculated as 100 ms/count, giving a maximum resolution of 10 Hz.

**Figure 3:**
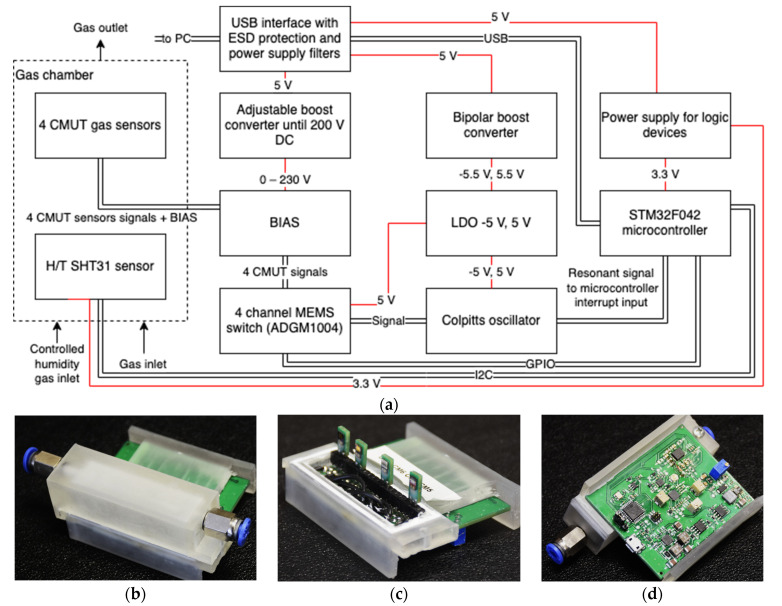
(**a**) Functional diagram of the sensor electronics. Overall view of the sensor: (**b**) PCB bottom view; (**c**) top view, cover off, and CMUTs exposed; (**d**) top view and cover on. The schematic and photographs are reproduced from [33].

The data exchange and general power supply were maintained via the USB interface. A block diagram for the designed electronics and photographs of the assembled electronics setup with 4 CMUT sensors are presented in Figure 3. The CMUT devices were driven by applying a 50 V bias and ±5 V sinusoidal AC. The CMUT membranes functioned in a non-collapse mode.

### 2.5. Raman Spectroscopy

The freshly synthesized ZIF-8 was drop-coated onto a gold surface and dried out in the atmosphere. A confocal WITec alpha300R microscope with a 532 nm laser and an ×100 objective was used to perform Raman spectroscopy on the ZIF-8 crystals. The spectra were taken from a couple of selected spots, starting from the sample surface. A depth profile was produced using a 0.5 µm step until a depth of −20 µm from the surface was reached.

### 2.6. Experimental Procedure

Typically, 4 differently coated CMUT sensors were simultaneously used during the experiments: (1) an unmodified sensor, which was used as a reference for any measurement drift compensation examples over time; (2) a sensor modified with the AZ photoresist solution, which was used as a reference for the compensation of the influence of the photoresist material on gas absorption; (3) a sensor modified with AZ photoresist mixed with ZIF-8, which was the main device for experimentation; and (4) a sensor modified with GOx, which was used as a reference to compensate for the changes in humidity due to its innate sensitivity to water vapor. The Gox-modified CMUT sensor parameters were previously researched and characterized in our previous work [33]. The preparation of the experiments followed this protocol:

- The gas line was flushed with N_2_;
- The temperature was stabilized (N_2_ was flushed until the temperature fluctuations over time decreased to <1 °C);
- The humidity stabilized (N_2_ was flushed until the humidity fluctuations over time decreased to <1% relative humidity (RH)).

**Figure 4:**
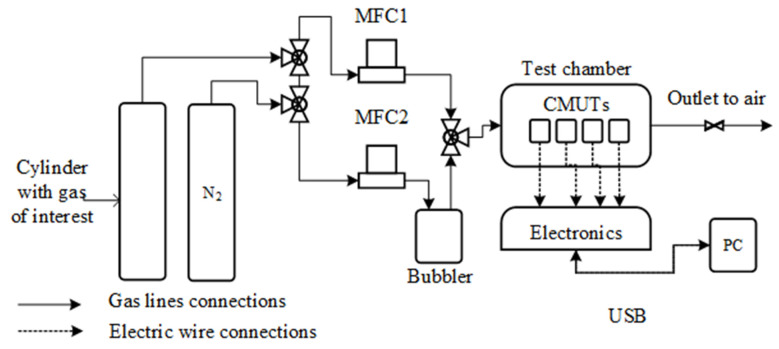
Principal schematic of the experimental setup with gases. The cylinder with the gas of interest was replaced with a gas cylinder of either methane, a butane/propane mixture, acetylene, or argon. For experiments with higher alkane vapors, the second tank with the gas of interest was removed and N_2_ gas was passed through the bubbler containing the selected alkane or mixture. The second channel contained pure N_2_ for comparison. For experiments with dry gases, the bubbler was either removed to produce dry gas or present and filled with H_2_O to produce H_2_O-saturated gas.

The butane/propane mixture was 70% propane/30% butane with trace amounts of thiolic compounds, as indicated by the producer. Other gases were pure, with at least 99.6% purity. To ensure a full gas exchange in the gas chamber, the gas chamber was designed to be as small as possible (5 cm^3^) and the fastest possible flow rates were used (unless deliberately changed in certain experiments). Using commercial CO_2_, humidity, and our previously designed GOx-based sensors, we ensured copious gas flushing for each experiment, with virtually all the gases being exchanged in dozens of seconds. Any response of the tested sensors past this point was considered to be a response rate of the sensor itself to the selected and fully tested chamber-saturated gas. The principal schematics of the experimental setup for humidity with saturated gases and alkanes are shown in Figure 4. After the initial preparation, devices were exposed to selected gases for selected times by programming the MFCs.

## 3. Results

### 3.1. Raman Spectroscopy of Synthesized ZIF-8

**Figure 5:**
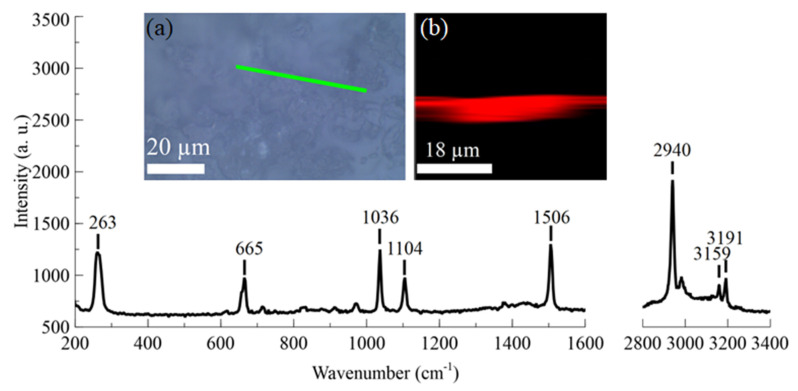
Raman spectroscopy results of ZIF-8 deposited on a gold surface; inset (**a**) micrograph of ZIF-8 crystals deposited on a gold surface (green line shows the depth scan location); inset (**b**) extracted principal component using the TCA (true component analysis) of ZIF-8 depth scan spectrum.

Raman spectroscopy was performed on dried-out ZIF-8 crystals deposited on a gold surface (Figure 5a). The observed peaks from highest to lowest were 263, 665, 1036, 1104, 1506, 2940, 3159, and 3191 cm^−1^. These could be assigned to ν(Zn-N), immidazolium ring puckering, ν(C5-N), ν(C5-N), ν(C4–C5), ν_asym_(C-H, methyl group), or ν(C-H, aromatic group), where ν denotes the stretching mode, asym is asymmetric vibration, and C5 and C4 are the adjacent carbon atoms in the imidazole ring structure. The data were in good agreement with previous ZIF-8 Raman characterization studies [34,35].

### 3.2. Gravimetric Response in Repeated Switching between Butane/Propane Gas Mixture and N_2_ Gas

**Figure 6:**
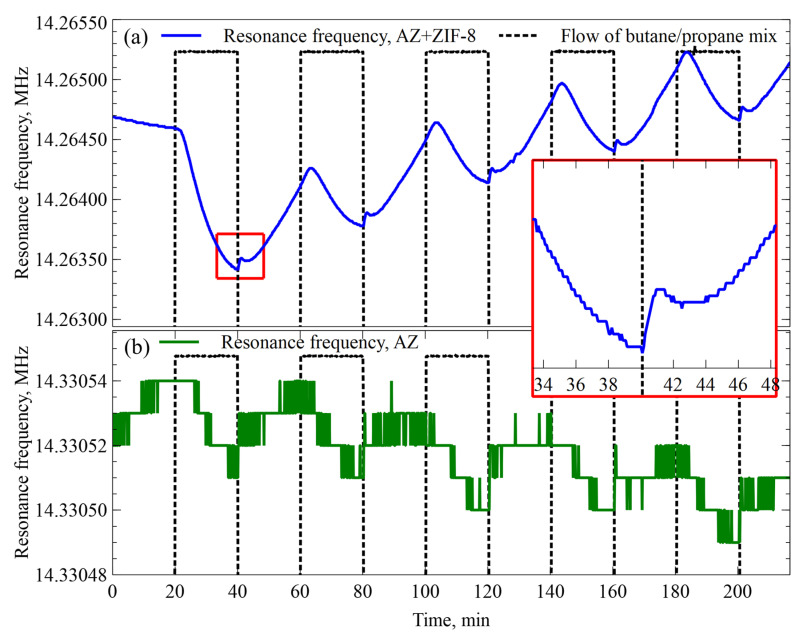
(**a**) The resonance frequency shift of the AZ+ZIF-8 resonator. The dashed line represents the flow rate of the butane/propane gas mixture. When the butane/propane gas mixture flow was turned off, humidity-saturated N_2_ gas was passed through. The inset shows further details of the resonance frequency shift when transitioning from the butane/propane gas mixture to N_2_ gas for the AZ+ZIF-8 resonator. (**b**) Resonance frequency shift of the AZ resonator.

AZ- and AZ+ZIF-8-coated resonators were exposed to humid N_2_ gas and a humid butane/propane gas mixture. Both resonators were observed during the same experiment; they were in the same test gas test chamber and connected to the same electronics setup. Both gases were exposed to the same humidification chamber. The data were acquired through two independent channels for each resonator. During the experiment, the butane/propane mixture and N_2_ gases were repeatedly swapped for each other, with each set of butane/propane gas mixture exposures and N_2_ gas exposures considered to be 1 cycle. Before the first cycle, the system baseline was established by allowing the control electronics temperature to stabilize and by flowing humid N_2_ for 10 min. The butane/propane mixture and N_2_ gases were introduced and changed in steps at a 12 sccm flow rate each and swapped between each other every 20 min. This was repeated for 5 cycles. The gathered data for the AZ+ZIF-8 resonator are shown in Figure 6. A zoomed-in version of a typical transition period between the butane/propane mixture and N_2_ is shown in the Figure 6 inset.

**Figure 7:**
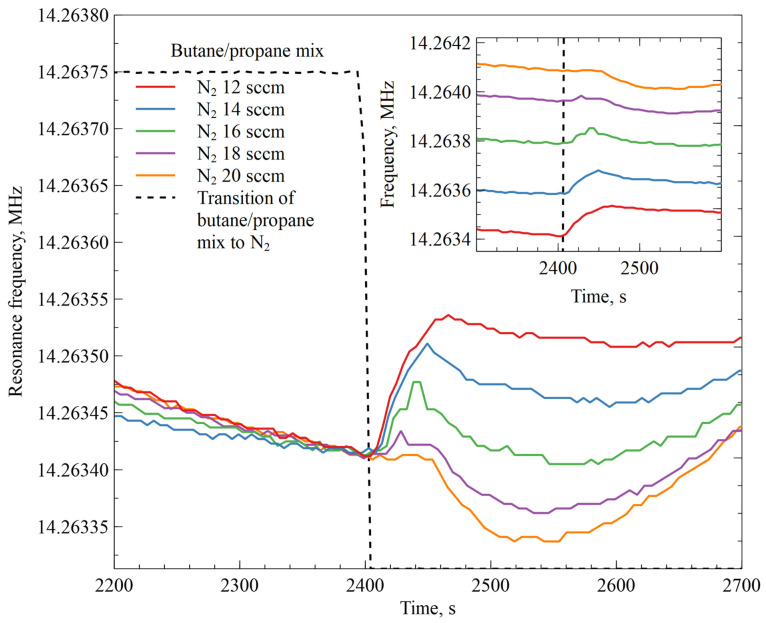
Resonator modified with AZ+ZIF-8 resonance frequency changes when transitioning between a humidity-saturated butane/propane mixture to humidity-saturated N_2_. The graphs are overlaid at the transition point and the average shift of resonance frequency was 170 Hz. For clarity, the inset shows a resonator modified with AZ+ZIF-8 resonance frequency changes when transitioning between a humidity-saturated butane/propane mixture to humidity-saturated N_2_ with an increasing flow rate for each step.

The transitory resonant frequency shift effects of the AZ+ZIF-8 resonator were further investigated by modifying the parameters and conducting an experiment. The results are depicted in Figure 7. In this case, the N_2_ gas flow was 12 sccm in the first cycle and was increased by 2 sccm with each cycle. The results are given in Supplementary Figure S2. The data and corresponding graph cutouts for each butane/propane mixture to N_2_ gas-switching event were pasted together to generate a single figure by overlapping each part of the graph aligned by the transition from the butane/propane mixture to N_2_.

### 3.3. Difference between Ar and N_2_ Gravimetric Responses

AZ- and AZ+ZIF-8-coated resonators were exposed to humid Ar and N_2_ gases. Both resonators were observed during the same experiment; they were in the same test gas test chamber and connected to the same electronics setup. The data were acquired through two independent channels for each resonator. Both gases were exposed to the same humidification chamber. During the experiment, Ar and N_2_ gases were repeatedly swapped for each other, with each set of Ar gas exposure and N_2_ gas exposure considered to be 1 cycle. Before the start of the first cycle, the system baseline was established by allowing the control electronics temperature to stabilize and by flowing humid N_2_ for 10 min. Ar and N_2_ gases were introduced at a 90 sccm flow rate each and swapped between each other every 20 min. This was repeated for 5 cycles.

**Figure 8:**
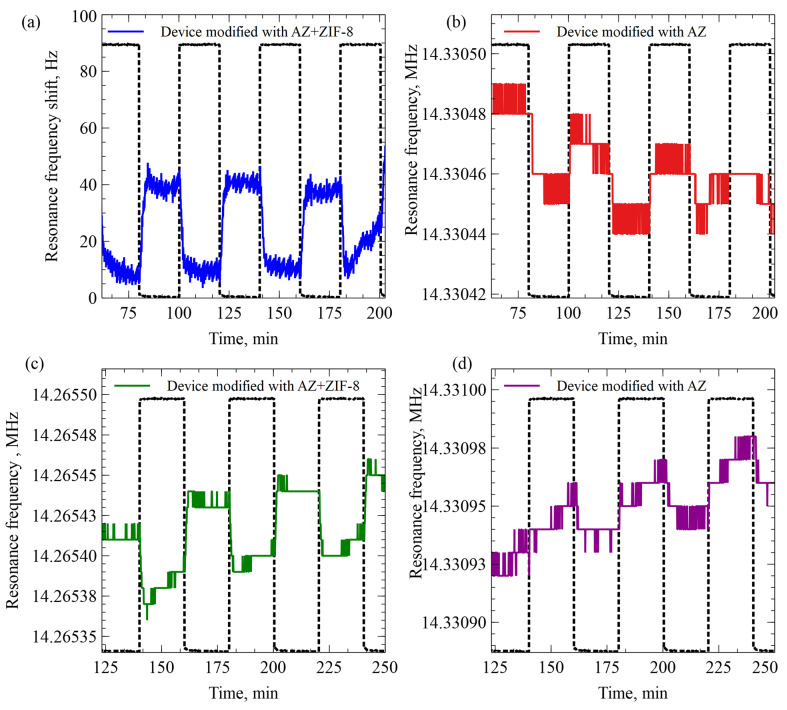
(**a**) The resonance frequency shift of AZ+ZIF-8 resonators when repeatedly exposed to Ar and N_2_ gases after subtracting the long-term frequency trend. When the Ar gas flow was turned off, humidity-saturated N_2_ gas was passed through at 90 sccm. (**b**) The resonance frequency shift of the AZ resonator when repeatedly exposed to Ar and N_2_ gases. When the Ar gas flow was turned off, humidity-saturated N_2_ gas was passed through at 90 sccm. (**c**) Change in resonance frequency of the resonator modified with AZ and ZIF-8 when Ar and N_2_ gases were humidity-desaturated. (**d**) Change in resonance frequency of the resonator modified with AZ photoresist when Ar and N_2_ gases were humidity-desaturated.

The AZ+ZIF-8-modified resonator exhibited a prolonged resonance frequency decrease throughout the experiment that did not correlate with the gas-switch action. For the easier data visualization of the Ar–N_2_ gas-switching effects, this long-term shift was canceled out using a linear trend line from the last 3.5 cycles of the data using the equation y = −0.030529 Hz/s x + 14,266,069.4975 Hz. The resulting data are shown in Figure 8a. The original data graph with no long-term resonance frequency decrease cancellation for this experiment is available in Supplementary Figure S1.

The AZ-modified resonator data are shown in Figure 8b. The resonator did not show any long-term effects as with the AZ+ZIF-8 resonator, so no trend-line cancellation was needed. Similarly, an experiment with dry gases was conducted; the data are given in Figure 8c,d. In this case, no trend-line cancellation was used either. Only AZ+ZIF-8 and humidity-saturated gas-switching demonstrated a significant response of a 30 Hz resonance shift between the N_2_ and Ar gas flows. The remaining three experiments showed noise-level gas-switching detection abilities.

### 3.4. Side-by-Side AZ+ZIF-8 and AZ Sensor Comparison in Response to Various Gases

**Figure 9:**
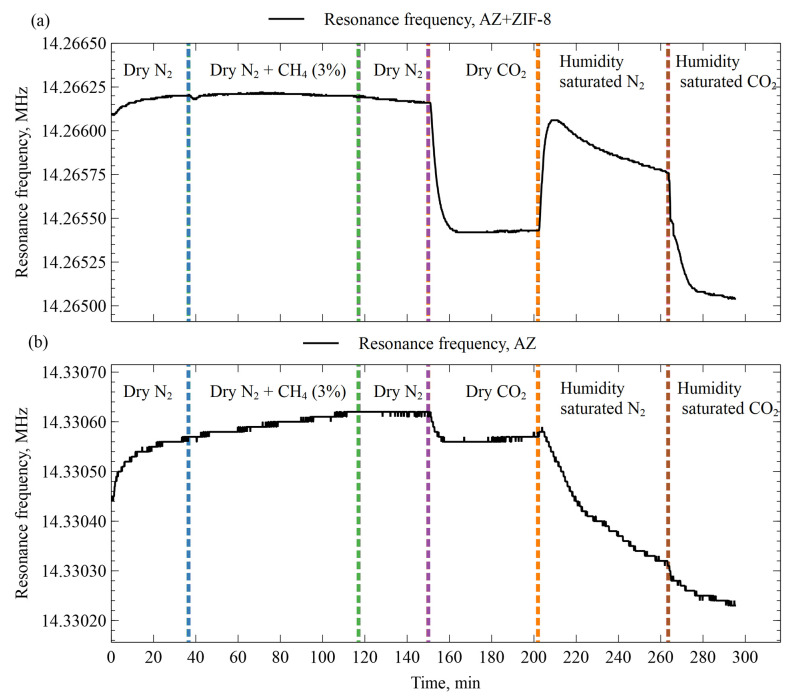
Resonance frequency shifts in AZ- and AZ+ZIF-8-modified resonators when exposed to a series of different gases. Dry N_2_ + CH_4_ (3%) indicates a gas mixture of 97% N_2_ and 3% methane. Colored dashed vertical lines indicate gas-switching instances. (**a**) AZ+ZIF-8 resonator frequency shifts. (**b**) AZ+ZIF-8 resonator frequency shifts.

To find out how the AZ+ZIF-8 and AZ-modified resonator resonance frequencies changed over time when different types of gases were introduced, two sensors were exposed to a series of gas flows. The data are shown in Figure 9a and Figure 9b, respectively. In both cases, there was no discernible response to a 3% methane addition to the N_2_ gas. Both sensors exhibited a marked response to dry CO_2_ gas, albeit with different levels. The AZ resonator had mass dampening in the range of tens of Hz, whereas the AZ+ZIF-8 resonator was affected up to 0.75 kHz. Humidity-saturated N_2_ affected both of the sensors, although in a different manner. The AZ sensor showed the largest mass-dampening effect by far of all the gases it was exposed to in this experiment, whereas the AZ+ZIF-8 sensor resonance frequency recovered to higher levels by 0.5 kHz. Finally, humidity-saturated CO_2_ also exhibited mass dampening for both of the sensors on similar levels as the dry CO_2_. Overall, the AZ+ZIF-8 device had a stronger response to any of the gases when compared with the AZ resonator.

### 3.5. AZ+ZIF-8 Resonator Response to Various Alkane Vapors

**Figure 10:**
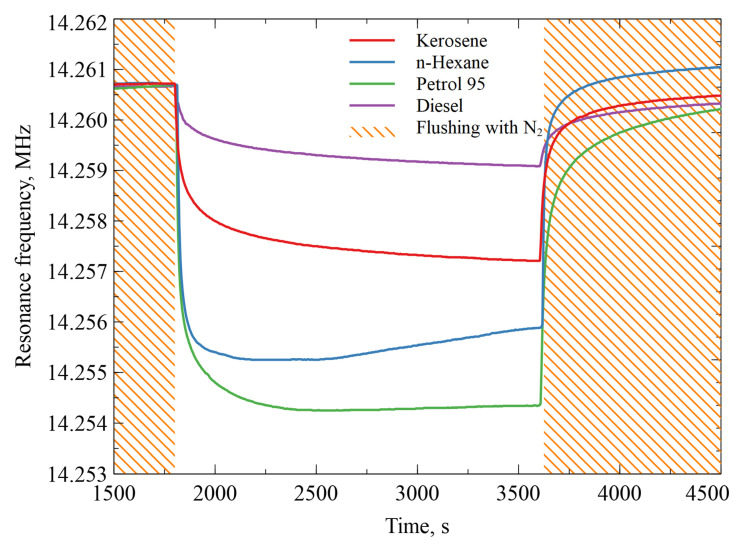
Resonance frequency shifts over time of the AZ+ZIF-8-modified resonator after a specific alkane vapor was introduced into the testing chamber.

The resonators modified with AZ+ZIF-8 were subjected to kerosene, diesel, gasoline 95, and n-hexane vapors where N_2_ was bubbled through the alkane liquids in a bubbler system, creating a constant flow of alkane-saturated gases that were introduced into the testing chamber. All experiments were first flushed with dry N_2_ until the change in temperature and relative humidity stabilized. Then, a constant flow of dry N_2_ gas was bubbled through the bubbler filled with the alkane liquid for about 0.5 h. The N_2_ flow rate was set, depending on each alkane, to maintain a 90 sccm flow rate for that specific vapor. The resonance frequency changes observed for each alkane are shown in Figure 10. Diesel fuel vapor had the smallest observable mass dampening of 1.8 kHz and gasoline 95 had the largest response of 6.2 kHz. The initial recovery positions of the resonance frequency following the subsequent N_2_ flushings were not identical to the pre-alkane flows, but did not deviate by more than 1 kHz and eventually stabilized back to the same initial value in a period of 16 h.

### 3.6. Full Hydrocarbon Response Comparison

**Figure 11:**
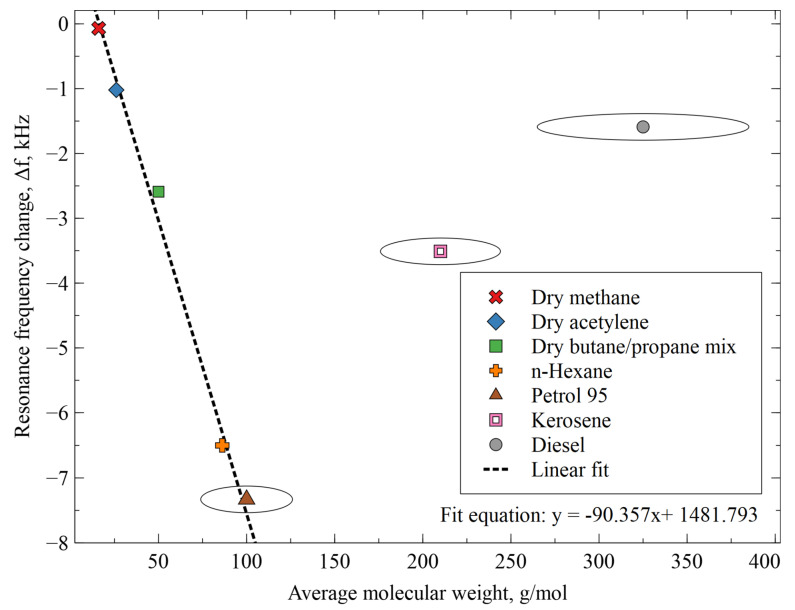
The resonance frequency shift of AZ+ZIF-8-modified resonator as a function of the average molecular weight of the tested dry gases and N_2_-based vapors. For higher hydrocarbons, average molecular weight was used for plotting. Ellipses depict the possible range of that specific hydrocarbon. The linear fit was used for the methane–gasoline 95 range. For gasoline 95, the average molecular weight was used for calculations.

Additional experiments on the AZ+ZIF-8 response to lower degree hydrocarbons—namely, methane, acetylene, and a butane/propane mixture—were conducted in a manner similar to the previous section, except no bubbler system was used and the dry gases flowed at a 90 sccm rate for 0.5 h. Pre-experiment and post-experiment dry N_2_ gas flushes were performed for 0.5 h as well. After the data were collected, they were plotted together with the higher alkane data from the previous section as the average molecular weight of the gas/vapor against the observed resonance frequency shift (Figure 11). There was a clear trend of a decreasing resonance frequency shift for gasoline 95, with a linear dependence on the molecular weight of the gas/vapor; the trend was reversed with kerosene and diesel. Whereas gasoline 95 reached a maximum frequency shift of 7.5 kHz, kerosene reached only 3.5 kHz and diesel reached only 1.5 kHz.

### 3.7. Gas Sensitivity Dependence on AZ+ZIF-8 Layer Thickness

**Figure 12:**
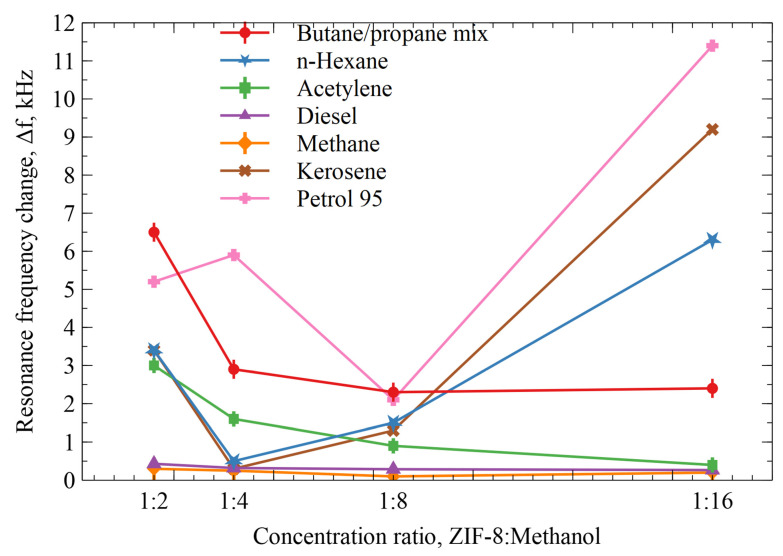
Resonance frequency change of the resonators modified by different ratios of AZ+ZIF-8 and methanol.

A similar experiment as before was conducted with the same hydrocarbons, but with a varying functional layer. Multiple resonators were functionalized with varying AZ+ZIF-8:methanol solutions with different dilutions of AZ+ZIF-8 and methanol, ranging from 1:2 to 1:16. The methanol evaporated, leaving a reduced-thickness AZ+ZIF-8 layer. The coating thickness was measured with a micrometer device. The measured average thicknesses for each methanol dilution sample were as follows: 1:2—83 µm; 1:4—56 µm; 1:8—12 µm; and 1:16—5 µm. The same pre-experiment and post-experiment flushings were performed, as previously described. The results of the resonance frequency change due to various deposited concentrations of ZIF-8 are given in Figure 12. The resonators modified at a 1:16 ratio exhibited an excellent response to gasoline 95, kerosene, n-hexane, and the butane/propane mixture. The highest dilution resonator also proved to be most sensitive to the molecular weight of the gas/vapor. As in previous results, differently functionalized resonators with AZ+ZIF-8:methanol ratios ranging from 1:2 to 1:16 showed the best resonance frequency shift responses of 7 to 11 kHz towards molecules with an average molecular weight of 100 g/mol, which were gasoline 95 and n-hexane. Kerosene had the highest sensitivity when exposed to the highest AZ+ZIF-8-concentration resonator.

## 4. Discussion

The sensing mechanism of the built devices was composed of two parts: the physicochemical interaction of the gas and vapor absorption into the ZIF-8 molecule cage structure or interstitial sites; and the mass loading of the CMUT membrane by the absorbed gas, which increased the dampening of the membrane and shifted the resonant frequency of the oscillating membrane to lower values. AZ was used as a carrier matrix to suspend and hold the ZIF-8 particles. Even though the data showed that AZ alone did interact with some of the gases, the interaction was on a lower order-of-magnitude scale than the AZ+ZIF-8 devices. This interaction might not even have been of chemical nature, with a trivial flow rate and pressure variations being compelling explanations. Therefore, we concluded that ZIF-8 dominated gas absorption, retention, and release, as described multiple times in previous research.

To decrease any unnecessary mass loading on CMUT membranes, initial CMUT sensors were drop-coated with methanol-suspended ZIF-8 (no AZ present). After drying out the methanol, these ZIF-8-only sensors were found to be unstable. The device resonance frequency and resonance amplitude markedly changed in minutes; optically, the observed ZIF-8 crystal structures on the surface reverted to an amorphous state, as it was before the methanol was fully dried out. Therefore, the AZ photoresist polymer was selected as an embedding matrix for ZIF-8 due to its solubility in methanol and compatibility with gold and silicon surfaces.

Additionally, AZ+ZIF-8 exhibited a moderate level of agglomeration whilst methanol evaporated after AZ+ZIF-8 was drop-coated onto the sensor surface, as visible in Figure 2d. This, we believe, significantly contributed to the thickness variability in the experiments described in Section 3.7 as well as splitting the resonance peak, where one major peak represented the main ZIF-8-populated area of the sensor and the rest arose from the thickness variability and uneven distribution of ZIF-8 particles. We provide an example of a resonance frequency split peak in Supplementary Figure S4.

ZIF-8 was found to be chemically stable over time, in agreement with previous research. Any sensor functionalization with freshly prepared, 1-day-old (kept in methanol), 1-week-old, and 1-month-old ZIF-8 showed similar responses and trends to all the gases. Supplementary Figure S5 demonstrates the repeatability of experiments after 1 week using the same sensors with selected gases. Therefore, any discrepancies in the results could be accounted for by the uneven thickness and distribution of the functional layer, produced by drop-coating the sensor surface (this was especially relevant for the gas sensitivity dependence on the layer thickness measurements with regards to the sample with the 1:8 AZ+ZIF-8 concentration as this seemed to underperform when compared with other samples). Similarly, the sensors that were functionalized with AZ+ZIF-8, used, and put aside for a week or a month exhibited the same behavior for any gases tested, with no deterioration in performance.

The control sensor functionalized with only the AZ photoresist demonstrated minimal sensitivity only to water vapor and CO_2_, which could easily be compensated for when constructing a commercial sensor using various known techniques. Curiously, the opposite response of AZ+ZIF-8 to humidity-saturated N_2_ compared with the AZ sensor might indicate that humidity compensation might not be needed in some cases. An AZ-modified sensor; a graphene-oxide-modified sensor; and another bare, unmodified sensor were used throughout the experiment series, including hydrocarbons. Their data were not shown for these experiments because they had no significant response at all, except for the graphene-modified sensor, which indicated a response to water vapor and CO_2_ (the research for this is published in another paper [33]).

The lack of a figure for CH_4_ gas testing was explained by no sensor response observed when switching from N_2_ to CH_4_ and back. This was unusual because the CH_4_ absorbance of ZIF-8 is well-documented and already exploited [18,23,36,37]. However, this work did not detect any reasonable response with regards to dry N_2_ to CH_4_, wet N_2_ to CH_4_, or even air to CH_4_-switching compared with AZ-coated and bare CMUT sensors. Considering that the CH_4_–ZIF-8 interaction strength of 10 kJ/mol [36] is of the order of a typical H···N hydrogen bond and CMUT-based sensors with H_2_O and CO_2_ with a similar bond strength bound to other functional materials have already been demonstrated [25,31,38], the vibrational environment could be a suitable explanation for such results. The ZIF-8 cage aperture size was 0.34 nm. CH_4_ was found to freely diffuse through ZIF-8 [39,40], demonstrating a loose methane coupling to the framework and, therefore, a low inertial mass component in the system of the adsorbed gas. The increased size of the higher hydrocarbons improved the mass loading of the target gas molecule, restricting its movement and creating more interaction points with the cage walls, therefore increasing the effective inertial mass loading onto the system and sensor response, as seen in Figure 10 and Figure 11.

However, the Ar–N_2_ gas-switching experiment demonstrated that weak binding molecules/atoms could still interact with ZIF-8 in such an environment. The comparative response of the test sensor to Ar and N_2_ was relatively small when compared with higher-order hydrocarbons, yet it was noticeable and reproducible. Ar has already been shown to occupy the ZIF-8 cage by filling it with several atoms [41]; the difference in sensor responses could be attributable to the number of molecules and their weight difference in the functional material.

Notably, the majority of ZIF-8 research stops at C3 or C4 hydrocarbons, with bigger molecules typically shown as having low self-diffusivity values [37]. Here, we present data showing an improved sensor response for gasoline 95 (~C8) and an additional lowered response for diesel (~C12 average, and up to C25 as the upper limit). The unique combination of AZ and ZIF-8 might play a role in enhanced diffusivity and subsequent sensor sensitivity; however, more research is needed to confirm this.

Additionally, AZ+ZIF-8 composition and layer formation research has the potential to drastically improve its sensitivity to hydrocarbons, add the functionality of hydrocarbon fractionation, and possibly improve the sensor response time due to lower depth penetration when the layer thickness is decreased.

The butane/propane mixture experiment demonstrated that potentially specific transitory effects could take place when the gases were switched. The effect was relatively fast (tens of seconds) and only observed with the butane/propane mixture and N_2_ switching. It could be attributable to the commercial use of butane/propane mixture additives such as thiolic compounds or a lack of fractional cleanliness, or it could be due to more complex mechanisms such as blocking layer formation when gases adsorb or desorb from the surface.

Additionally, the observed sensor drifts in a few cases that are given in Supplementary Figures S1 and S2 were not fully investigated, but they did not impact the overall direct responses to gas changes.

The drift was observed only in resonators exposed to Ar and butane/propane mixture gases. These results were reproducible with different sensors functionalized the same way and in repeated experiments using the same sensors. Considering that Ar is a noble gas and that the sensor resonant frequency recovered to initial values before the next experiment, the more likely explanation for such drifts is physical, involving gas absorption kinetics rather than chemical and based on gas reactions with the sensing layer or the chemical degradation of the sensing layer.

Our previous work on Gox-modified CMUT resonators showed their suitability as humidity sensors [32]. In this work, these sensors were used as an additional reference channel for humidity monitoring throughout most of the experiments. In addition to their response to water vapor, the GOx sensors exhibited a small response to the butane/propane mixture and argon gas-switching. The data are presented in Supplementary Figure S3. Considering the small response and the fact that the ZIF-8-functionalized sensor was the main research topic, the GOx response to non-H2O vapor was considered to be irrelevant for this research.

## 5. Conclusions

The manufactured gravimetric CMUT sensors functionalized with an AZ+ZIF-8 compound demonstrated measurable resonance frequency shifts for various gases, including C3–C25 alkanes and acetylene. This was a unique finding because no other ZIF-8 sensor has been made for such heavy hydrocarbons to the best of our knowledge. The influence of the AZ photoresist polymer was found only on mass loading and the response to water vapor, which could be easily controlled. Contrary to previous findings, the alkane gas interaction with ZIF-8 increased up to about C8 alkanes and then decreased for higher-weight molecules in this unique vibrational environment. This widens the previously known useful ZIF-8 interaction range, which might apply to other ZIF-8 uses such as gas storage and gas separation. The observed resonance frequency shifts had a strong linear correspondence with the molecular weight of the alkane or alkyne used. Further layer deposition optimizations would be beneficial because thinner ZIF-8 layers demonstrated higher sensitivity to the gas molecular weight.

The demonstrated CMUT sensor has potential as a safety or fractionation monitoring device in hydrocarbon-based industries such as mining, oil refining, and natural gas extraction.

## Data Availability

The data presented in this study are available upon request from the corresponding authors.

## Supplementary Materials

The following supporting information can be downloaded at: https://www.mdpi.com/article/10.3390/s23218827/s1, Figure S1: Long-term resonance frequency shift observed in Ar and N_2_ environments for the AZ+ZIF-8 sensor; Figure S2: Long-term resonance frequency shift observed in humidity-saturated N_2_ and butane/propane mixture environments for the AZ+ZIF-8 sensor. The graph is color-coded for different graph sections, which are overlapped in Figure 7; Figure S3. Resonance frequency shift observed for the CMUT device functionalized with GOx during experiments (a) with a butane/propane mixture (corresponding with Section 3.2 experiments); (b) with argon (corresponding with Section 3.3 experiments); and (c) when exposed to a series of different gases (corresponding with Section 3.4 experiments); Figure S4. A typical frequency spectrum of a CMUT device modified with an AZ+ZIF-8 functionalization layer. Figure S5. Resonance frequency response of AZ+ZIF-8 modified devices showing the negligible difference between the sensor response to gases after 7 days; (a) AZ+ZIF-8 modified device response to diesel gas; (b) AZ+ZIF-8 modified device response to kerosene gas; (c) AZ+ZIF-8 modified device response to argon gas.
