# Supplementary material for: Design of Zeolitic Imidazolate Framework-8-Functionalized Capacitive Micromachined Ultrasound Transducer Gravimetric Sensors for Gas and Hydrocarbon Vapor Detection
Source: Sensors (Basel). 2023 Oct 30;23(21):8827. doi: 10.3390/s23218827 (PMC10648066; doi:10.3390/s23218827)
Supplement: Supplementary file 1 [file sensors-23-08827-s001.zip › sensors-2624108-supplementary.pdf]

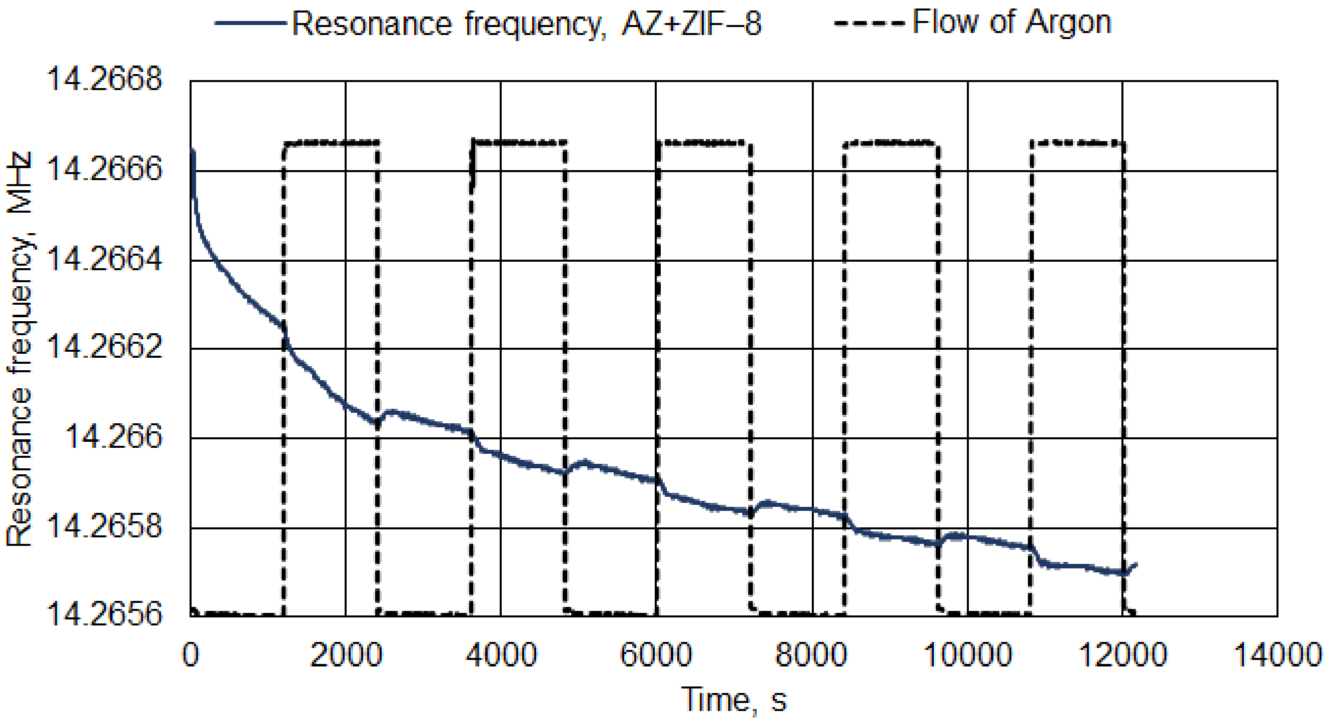

Figure S1. Long term resonance frequency shift observed in the Ar and N2 environments for the AZ+ZIF–8 sensor.

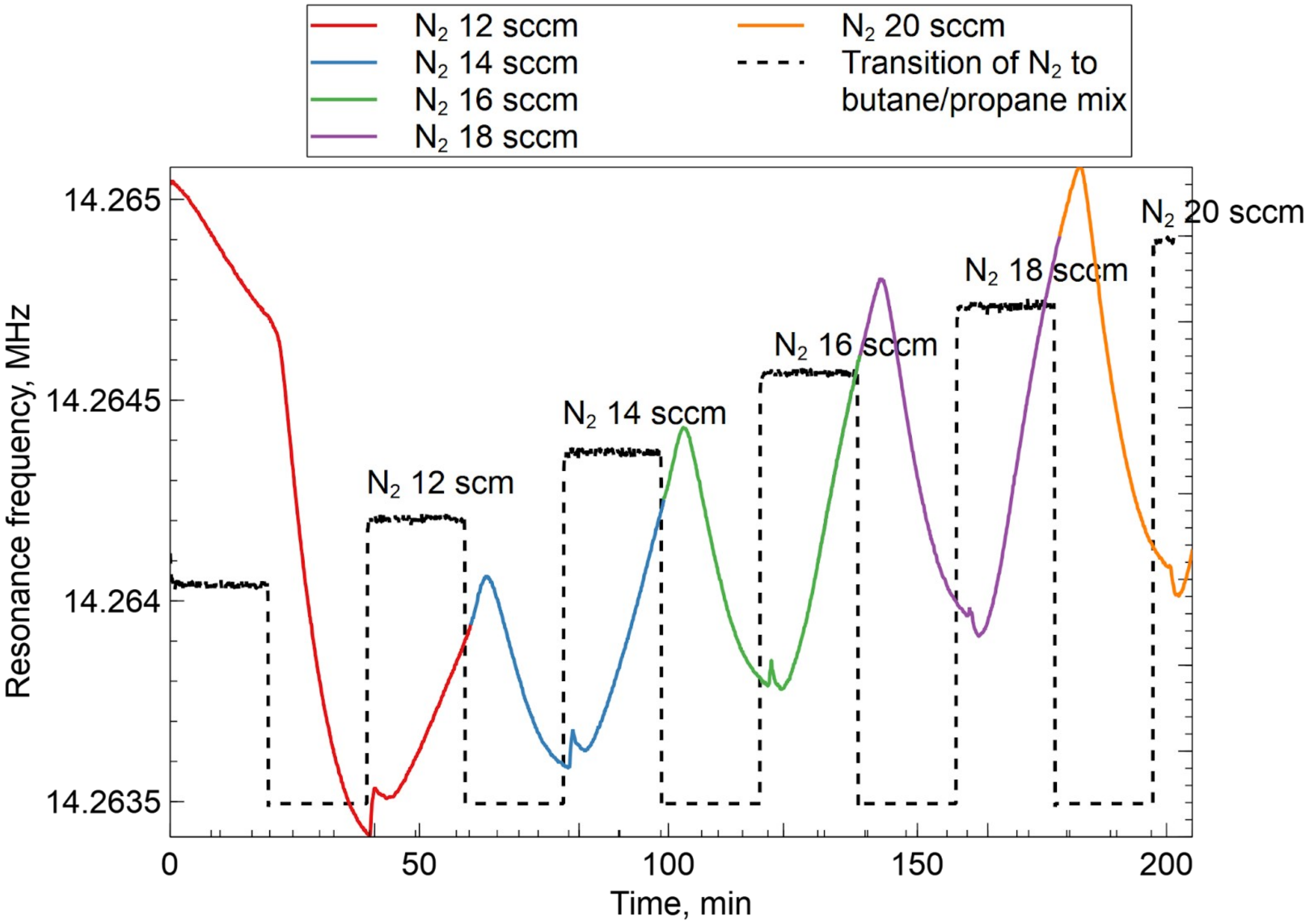

Figure S2. Long term resonance frequency shift observed in humidity–saturated N2 and butane/propane mix environments for the AZ+ZIF–8 sensor. Also, the graph is color-coded for different graph sections which are overlapped in figure 7.

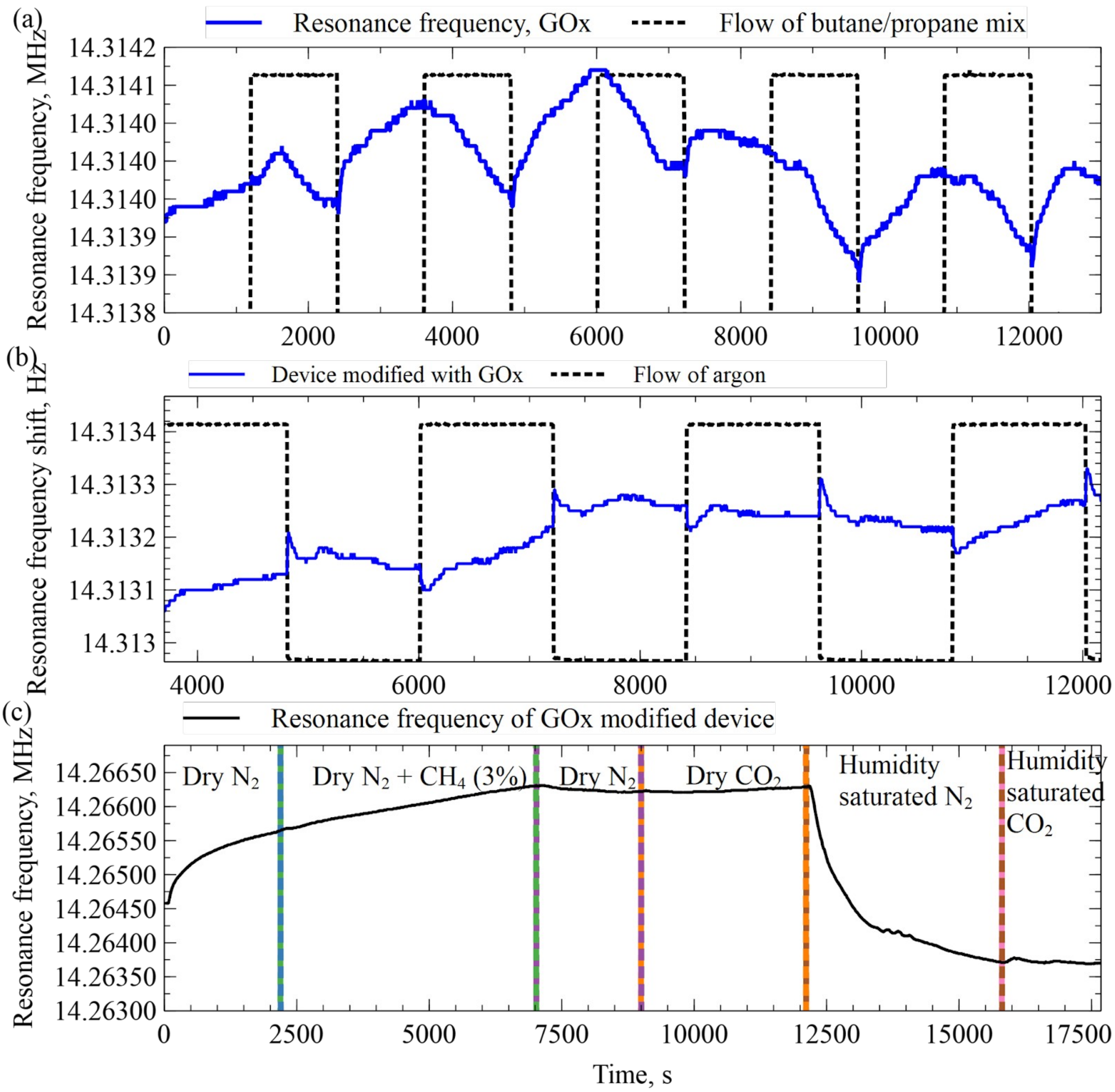

Figure S3. Resonance frequency shift observed for the CMUT device functionalized with GOx during experiments with (a) butane/propane mix (corresponds to main paper section 3.2 experiments; (b) argon (corresponds to section 3.3 experiments); (c) when exposed to a series of different gases (corresponds to section 3.4 experiments).

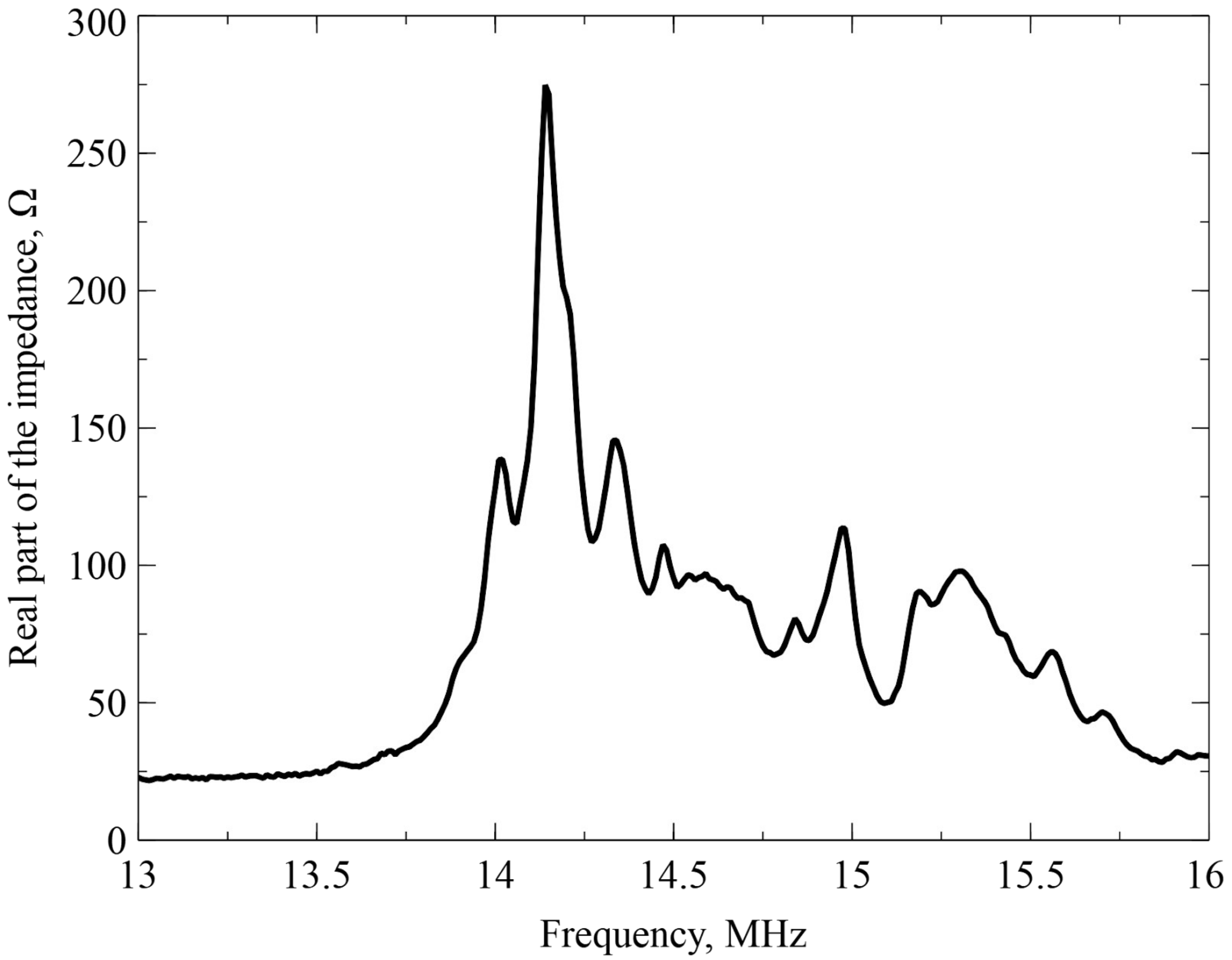

Figure S4. A typical frequency spectrum of a CMUT device modified with AZ+ZIF-8 functionalization layer.

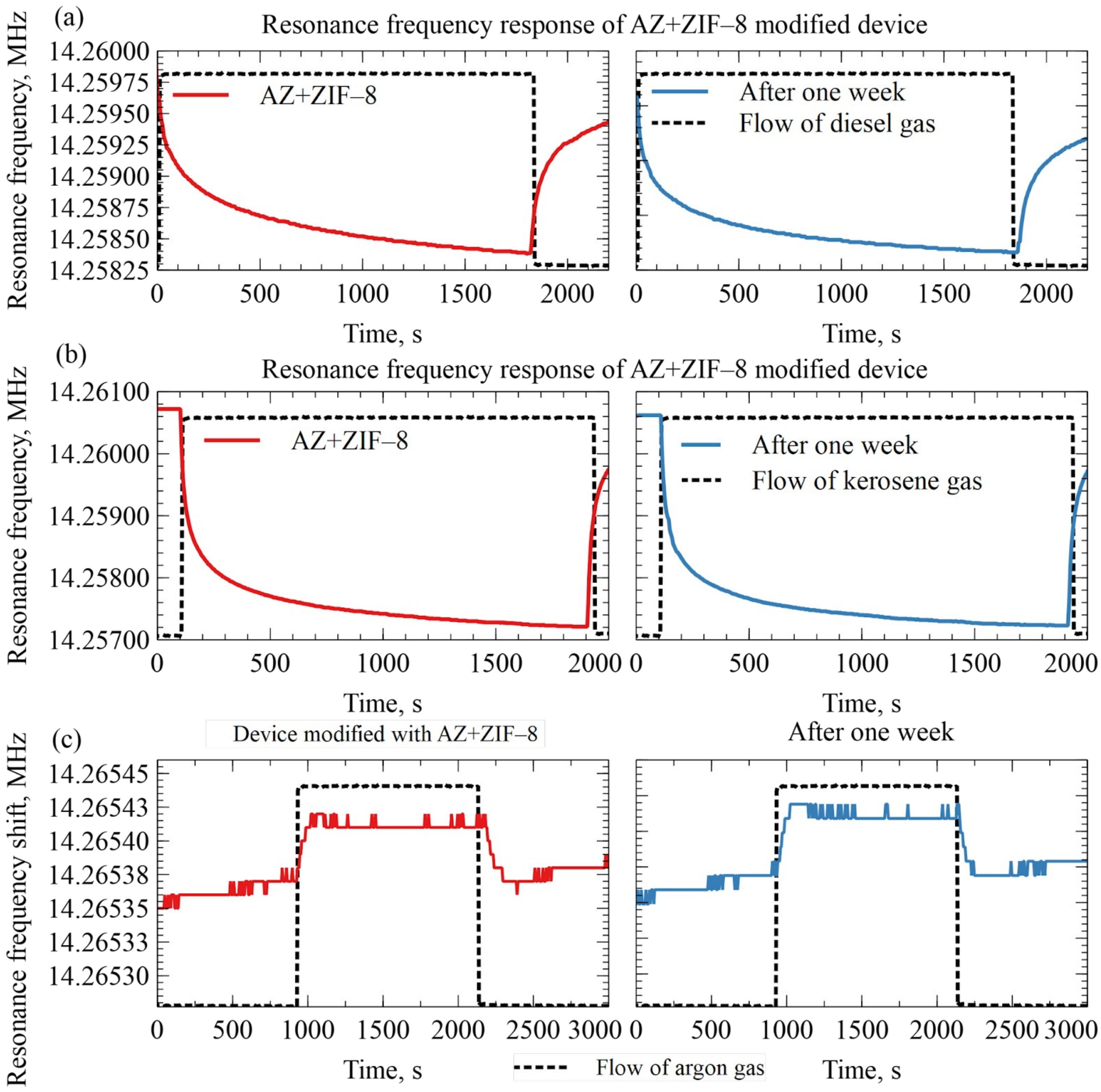

Figure S5. Resonance frequency response of AZ+ZIF–8 modified devices showing the negligible difference between the sensor response to gases after 7 days; (a) AZ+ZIF–8 modified device response to diesel gas; (b) AZ+ZIF–8 modified device response to kerosene gas; (c) AZ+ZIF–8 modified device response to argon gas.
